# Exosome‐mediated pyroptosis of miR‐93‐TXNIP‐NLRP3 leads to functional difference between M1 and M2 macrophages in sepsis‐induced acute kidney injury

**DOI:** 10.1111/jcmm.16449

**Published:** 2021-03-21

**Authors:** Chen‐Xia Juan, Yan Mao, Qian Cao, Yan Chen, Lan‐Bo Zhou, Sheng Li, Hao Chen, Jia‐He Chen, Guo‐Ping Zhou, Rui Jin

**Affiliations:** ^1^ Department of Pediatrics the First Affiliated Hospital, Nanjing Medical University Nanjing China; ^2^ Department of Nephrology, Affiliated Hospital of Nanjing University of Chinese Medicine Jiangsu Province Hospital of Chinese Medicine Nanjing China; ^3^ Department of Nephrology, Affiliated Geriatric Hospital, Nanjing Medical University Nanjing China; ^4^ Department of Dermatology the First Affiliated Hospital, Nanjing Medical University Nanjing China; ^5^ Department of Pediatrics Yancheng Maternity and Child Health Care Hospital Yancheng China; ^6^ Department of Urology the First Affiliated Hospital, Nanjing Medical University Nanjing China

**Keywords:** AKI, exosomes, macrophage, miR‐93, pyroptosis, TXNIP

## Abstract

Sepsis is a systemic inflammatory response syndrome caused by infection, resulting in organ dysfunction. Sepsis‐induced acute kidney injury (AKI) is one of the most common potential complications. Increasing reports have shown that M1 and M2 macrophages both take part in the progress of AKI by influencing the level of inflammatory factors and the cell death, including pyroptosis. However, whether M1 and M2 macrophages regulate AKI by secreting exosome remains unknown. In the present study, we isolated the exosomes from M1 and M2 macrophages and used Western blot and enzyme‐linked immunosorbent assay (ELISA) to investigate the effect of M1 and M2 exosomes on cell pyroptosis. miRNA sequencing was used to identify the different miRNA in M1 and M2 exosomes. Luciferase reporter assay was used to verify the target gene of miRNA. We confirmed that exosomes excreted by macrophages regulated cell pyroptosis in vitro by using Western blot and ELISA. miRNA sequencing revealed the differentially expressed level of miRNAs in M1 and M2 exosomes, among which miR‐93‐5p was involved in the regulation of pyroptosis. By using bioinformatics predictions and luciferase reporter assay, we found that thioredoxin–interacting protein (TXNIP) was a direct target of miR‐93‐5p. Further in vitro and in vivo experiments indicated that exosomal miR‐93‐5p regulated the TXNIP directly to influence the pyroptosis in renal epithelial cells, which explained the functional difference between different phenotypes of macrophages. This study might provide new targets for the treatment of sepsis‐induced AKI.

## 1 INTRODUCTION

Sepsis is a life‐threatening organ dysfunction caused by the host's dysfunctional response to infection,^1^ leading to multiple organ dysfunction syndrome including acute kidney injury (AKI).^2^ AKI is defined as a group of clinical syndromes that refers to a sudden (1‐7 days) and sustained (>24 hours) sudden decline in renal function.^3^ It has been alleged that 50% of AKI cases are triggered by severe sepsis, resulting in a 50%‐70% mortality in AKI.^4^, ^5^ The mechanism of sepsis‐associated AKI includes haemodynamic hypothesis and inflammatory factor hypothesis, but these hypotheses cannot explain all AKI included in the process of sepsis.^4^ The exact mechanism remains to be further verified.

AKI is characterized by damage or death of tubular epithelial cells, in which pyroptosis has been reported to play a role in the progression of AKI.^6^ Pyroptosis is a newly discovered mode of programmed cell death in inflammatory cells that mediated by the activation of various caspases, mainly caspase‐1, and the formation of inflammatory bodies.^7^ A variety of Gasdermin family members shear and multimerize during the process of pyroptosis, leading to cell perforation, which in turn causes cell death.^7^ Compared with apoptosis, pyroptosis occurs more rapidly and is accompanied by the release of a large number of pro‐inflammatory factors.^8^ Studies have discovered pyroptosis in glomerular epithelial cells and proximal tubule cells and declared that pyroptosis may be involved in the pathophysiological process of tubular epithelial cells injury in sepsis‐associated AKI.^9^, ^10^, ^11^

Macrophages (MФ) is one of the most important leucocyte species involved in AKI.^12^ Monocyte‐macrophages have been found to mediate the acute phase within the first 24 hours of AKI, promoting inflammatory cell infiltration.^13^ Actually, at different stages of injury, macrophages differentiate into different phenotypes and play different roles.^12^ In the early stages of AKI, the kidney tissue exhibits a ‘sterile inflammation response’. At this stage, the phenotype of macrophage is mainly M1, which releases the pro‐inflammatory mediator and causes damage to the proximal tubule of the outer layer of the renal medulla. As the disease progresses, macrophages gradually gather and engulf apoptotic cells or their released factors. Meanwhile, the phenotype transforms from pro‐inflammatory M1 macrophages to anti‐inflammatory M2 macrophages.^14^, ^15^ Therefore, we speculate that the M1 macrophages may be the ‘culprit’ that promotes the cell death of renal epithelial cells and eventually causes tubular necrosis, while M2 macrophages can alleviate tubular necrosis.

Exosomes are phospholipid bilayer vesicles derived from the endosome pathway, ranging from tens to hundreds of nanometres in diameter.^16^ They contain abundant protein and genetic information substances that mediate the exchange of substances between cells.^17^, ^18^ It was found that microRNAs (miRNAs) transmitted by exosomes can regulate as signal molecules. For example, monocytes‐derived exosomes transported miR‐150 to endothelial cells to promote migration of endothelial cells or angiogenesis.^19^ Studies also show that macrophage‐derived exosomal miRNAs play important roles in kidney diseases, including AKI.^20^, ^21^

This project intends to explore whether M1 and M2 macrophages transmit miRNA through exosomes to influence the pyroptosis in renal epithelial cells. The specific molecular mechanisms involved in the functional difference between M1 and M2 macrophages in AKI were elucidated.

## 2 MATERIALS AND METHODS

### 2.1 Cell culture

Primary peritoneal macrophages were harvested from the peritoneal exudates of 6‐ to 8‐week‐old BALB/c female mice following the established protocol.^22^ Briefly, 72 hours after injecting 2 mL of 3% proteose peptone per mouse into the peritoneal cavity, mice were killed by rapid cervical dislocation. Then, peritoneal fluid was withdrawn slowly. After centrifugation, cells were cultured at 37°C in 5% CO_2_ in Dulbecco's modified Eagle medium (DMEM, Gibco, Grand Island, NY, USA) supplemented with 10% foetal bovine serum (FBS, Gibco), 100 U/mL penicillin, 100 µg/mL streptomycin and 20% L929‐conditioned medium, with the cell concentration adjusted to 2‐3 × 10^6^ cells/mL. After 1‐2 hours, non‐adherent cells were removed by washing with PBS, and adherent cells left were uncommitted macrophages (MФ). TCMK‐1 cells, a mouse kidney epithelial cell line (CCL‐139), were purchased from the American Type Culture Collection (ATCC) and incubated in complete DMEM, at 37°C in 5% CO_2_. To mimic the inflammatory state, TCMK‐1 cells were stimulated with 100 ng/mL of lipopolysaccharide (LPS, Sigma‐Aldrich, Saint Louis, MO, USA) for 24 hours.

### 2.2 Cell transfection

Overexpression or knockdown of miR‐93‐5p in TCMK‐1 was performed by transfecting the miR‐93‐5p mimic or inhibitor (Ribo Bio, Guangzhou, China) at the concentration of 50 and 200 nM, respectively, according to the manufacturer's instruction. Overexpression or knockdown of miR‐93‐5p in macrophage was performed by infecting the cells with miR‐93‐5p overexpression or knockdown lentivirus (GenePharma, Shanghai, China). To generate TCMK‐1 overexpressing TXNIP (OE‐TXNIP), TXNIP was amplified by PCR. And the 5′‐primer was modified to introduce an EcoRV restriction site, while the 3′‐primer was modified to introduce an XhoI restriction site and to reconstitute a stop codon. The product was cloned into pAc 5.1/V5‐HisA (Life Technologies, Carlsbad, CA, USA), using the corresponding plasmids without cDNA served as controls. SiRNA duplexes targeting TXNIP and control siRNA were synthesized by Shanghai Genechem Co. After testing for knockdown efficiencies, the stem‐loop oligonucleotide was synthesized and cloned into the lentivirus‐based vector. A non‐targeting (scrambled) stem‐loop DNA vector was also generated as a negative control. Lentivirus‐delivered TXNIP‐siRNA was then transfected into cells. The sequences of miR‐93‐5p mimic, inhibitor and siTXNIP were as follows:

mmu‐miR‐93‐5p mimic: 5′‐CAAAGUGCUGUUCGUGCAGGUAG‐3′

mmu‐miR‐93‐5p inhibitor: 5′‐CUACCUGCACGAACAGCACUUUG‐3′

siTXNIP: 5′‐GGUGUGUGAAGUUACUCGUTT‐3′

siNC: 5′‐UUCUCCGAACGUGUCACGUTT‐3′

### 2.3 Polarization of M1 and M2 macrophages in vitro and identification

For inflammatory macrophage (M1) differentiation, MФ were stimulated with 100 ng/mL LPS and 100 ng/mL recombinant IFN‐γ (R&D Systems, Minneapolis, MN, USA) for 24 hours. To generate anti‐inflammatory macrophages (M2), MФ were stimulated with 100 ng/mL recombinant IL‐4 (R&D Systems), 20ng/mL recombinant IL‐10 (R&D Systems) and 100 ng/mL recombinant IL‐13 (R&D Systems) for 24 hours. Polarized cells were identified by flow cytometry with myeloid and lymphoid immunophenotyping panels. Primary antibodies used in the flow cytometry analysis are as follows: CD11b (BD Biosciences, Franklin Lakes, NJ, USA), CD206 (BD Biosciences) and F4/80 (Thermo Fisher Scientific, Waltham, MA, USA). Data were collected using a BD LSRFortessa analyser and analysed using FlowJo 10.0 software.

### 2.4 Layered co‐culture

The transwell co‐culture model was established based on the modification of a previously published method.^23^ In the present model, TCMK‐1 cells and different phenotypes of macrophages were co‐cultured on the bottom and top surface of transwell microporous membrane, respectively. Briefly, a transwell insert with microporous membrane was gently wrapped around the edge using the sterilized parafilm (Parafilm M®, Bemis, USA) to build a parafilm fence. TCMK‐1 cells were then plated on bottom side of transwell insert membrane and were incubated for over 6 hours in medium at 37°C in 5% CO_2_ to attach to the microporous membrane firmly. Next, the transwell insert was turned over and put back to allow TCMK‐1‐plated side to face down. Polarized macrophages were seeded onto the top side of membrane. Finally, the resultant transwell plate was incubated at 37°C in 5% CO_2_ in ECM. Thus, TCMK‐1 cells formed the first layer on bottom side of the membrane, while macrophages formed the second layer on top side. The layers were isolated by the membrane, but the medium could diffuse freely across the membrane.

### 2.5 Cell viability assay

Standard 3‐(4,5‐dimethylthiazol‐2‐yl)‐2,5‐diphenyltetrazolium bromide (MTT) assays were conducted to detect the cell viability. At 24 hours after co‐cultured, TCMK‐1 cells were seeded into 96‐well plates. MTT solution was added into the medium to treat cells for 4 hours. Then, the medium was removed softly, and 150 μL dimethylsulphoxide (DMSO, Sigma‐Aldrich) was injected to dissolve the formazan. The absorbance was measured at 570 nm ± 10 using a plate‐reader (Bio‐Rad Laboratories). The cell viability index is expressed as relative value of control group.

### 2.6 Enzyme‐linked immunosorbent assay

The concentrations of TNF‐α, IL‐12, IL‐10, IL‐18 and IL‐1β in medium and serum were determined using commercial ELISA kits (R&D Systems) following the manufacturer's instructions.

### 2.7 Isolation, characterization and analysis of exosomes

The treatment conditions for M1 and M2 macrophages polarization have been described previously. Subsequently, we collected exosomes from the supernatants of M1 and M2 cell cultures, respectively, without additional stimulation. Exosomes were isolated using ExoQuick (System Biosciences, Palo Alto, CA, USA) according to the manufacturer's instructions. Briefly, media were collected and centrifuged at 2000 g for 10 minutes at 4℃. To thoroughly remove cellular debris, media were centrifuged again at 10 000g for 30 minutes. Reagents were then added and the mixture was vortexed and put overnight at 4℃. After centrifuged at 1500 g for 30 minutes at 4℃, the pellet containing exosomes was resuspended in PBS or ultrapure water. The isolated exosomes were verified by TEM, particle analyser and specific protein markers. Exosomes were fixed in 2.5% buffered glutaraldehyde overnight at room temperature. It was subsequently stained by 1% osmium tetroxide for 2 hours. Then, exosomes were treated by gradient ethanol dehydration, embedded in resin and examined with a TEM. The exosome particle size and concentration were measured by nanoparticle tracking analysis (NTA) with ZetaView PMX 110 (Particle Metrix, Meerbusch, Germany). For cell treatment, 2 μg of EVs (equivalent to those collected from ~5 × 10^6^ M1 or M2 macrophages) was added to 2 × 10^5^ TCMK‐1 cells.

### 2.8 miRNA sequencing

The differentially expressed miRNA level in the exosomes was screened by BGI Genomics (Shenzhen, China), as previously described.^24^ Briefly, total RNA was isolated to prepare the libraries. After the single‐strand DNA circle (ssDNA circle) was made to construct the final miRNA library, the DNA nanoballs (DNBs) were loaded into the patterned nanoarrays and single‐end read of 50 bp were read through on the BGISEQ‐500 platform. To obtain miRNA profiles that differently distributed in M1 and M2 macrophages, we set the fold change threshold of M2 group at |log2 (Fold Change)| >1 as compared to M1 group for further analysis. The differentially expressed miRNAs were determined using ANOVA.

### 2.9 RNA extraction and qPCR

Total RNAs were extracted by using TRIzol reagent (Thermo Fisher Scientific) and were quantified using the NanoDrop ND‐1000 (Thermo Fisher Scientific).

According to the results of high‐throughput sequencing, specific complementary DNAs (cDNAs) were synthesized from 10 ng of RNA eluate using TaqMan MicroRNA Reverse Transcription Kit (Applied Biosystems, Foster City, CA, USA). Then, the quantitative real‐time PCR (qRT‐PCR) was performed by amplifying cDNA using TaqMan MicroRNA Assay Mix (Thermo Fisher Scientific) to confirm the expression level of the screened five microRNAs. Primers for qRT‐PCR used are shown as following:

**Table:** 

mmu‐miR‐17‐5p	F: 5′‐CAAAGTGCTTACAGTGC‐3′
	R: 5′‐GTGCAGGGTCCGAGGT‐3′
mmu‐miR‐93‐5p	F: 5′‐TGCTCAGGTAGTGGTTGTCG‐3′
	R: 5′‐CACATGAAGCAGCACGAC‐3′
mmu‐miR‐106b‐5p	F: 5′‐AATGCCGCACTGTGGGTACT‐3′
	R: 5′‐GTGCAGGGTCCGAGGT‐3′
mmu‐miR‐20a‐5p	F: 5′‐GCGGCGGTAAAGTGCTTATAGTG‐3′
	R: 5′‐TGCAGGGTCCGAGGTAT‐3′
mmu‐miR‐20b‐5p	F: 5′‐CAAAGTGCTCATAGTGC‐3′
	R: 5′‐TGTCGTGGAGTCGGCAATT‐3'
Gapdh F:	5′‐ATCAAGAAGGTGGTGAAGCGGAA‐3′
	R: 5′‐TGGAAGAGTGGGAGTTGCTGTTGA‐3′

### 2.10 Luciferase reporter assay

Cells were co‐transfected with plasmids containing 3′‐UTR of wild or mutant fragments and miR‐93 mimics or inhibitor using Lipofectamine 3000 (Invitrogen, Foster City, CA, USA). At 48 hours after co‐transfection, firefly and renilla luciferase activities in TCMK‐1 cells were detected consecutively using dual‐luciferase reporter assay system (Promega, MA, USA). The firefly luciferase activity was normalized by renilla luciferase activity. Each assay was repeated in three independent experiments.

### 2.11 Animals

A total of 60 female 8‐ to 12‐week‐old BALB/c mice (weighing 18‐22 g) were purchased for this study. All mice were reared in plastic cages and given free access to food and water under standard conditions—temperature 25 ± 2°C, humidity 55 ± 5% and 12‐hours light/dark cycle. The mice were randomly divided into six groups: a control group (abdominal injection of physiological saline), a 293T‐exosomes group, a M1‐exosomes group, a M1‐miR‐93‐OE‐exosomes group, a M2‐exosomes group and a M2‐miR‐93‐KD‐exosomes group. To establish a sepsis‐induced model, caecal ligation and puncture (CLP) was performed as described previously.^25^ In brief, mice were anaesthetized via intraperitoneal injection of chloral hydrate, after which a skin incision of approximately 15 mm in the midline abdominal area was performed under aseptic conditions to expose the caecum. Then, a single through‐and‐through puncture was performed by a 20‐gauge needle between the ligation site and the end of the caecum and a small amount of faecal material was extruded, allowing peritoneal dissemination of bacteria after the caecum was ligated by polyglactin sutures. The caecum was repositioned carefully into the peritoneal cavity, and the laparotomy was closed, followed by fluid resuscitation. The mice were killed 24 hours after CLP. Blood samples were collected, and kidney samples were harvested and stored at −80°C until further analysis. Serum creatinine (Cr) and blood urea nitrogen (BUN) were measured by using the Cr assay kit and BUN assay kit (Jiangcheng Bioengineering Institute, Nanjing, China). All experiments on animals followed the Guide for the Care and Use of Laboratory Animals, which was published by the Ministry of Health of People's Republic of China. The study was approved by the Ethics Committee of the Jiangsu Province People's Hospital.

### 2.12 Histological studies

The kidney tissue sections collected from mice were immediately fixed in 10% neutral‐buffered formalin and were paraffin‐embedded to generate tissue blocks. The tissue blocks were cut at 4 µm thickness. The sections were routinely dewaxed with xylene, washed with ethanol, and performed with haematoxylin and eosin staining (H&E) and periodic acid‐Schiff (PAS) staining as previously described.^26^ The pathomorphological changes of the renal tissues from each group were then observed under a microscope.

### 2.13 Western blotting

After harvested and centrifuged, cells were lysed in loading buffer, which was prepared as previously described.^27^ All samples including exosomes were boiled for 10 minutes before loading. Proteins were separated by SDS‐PAGE and then transferred onto the polyvinylidene fluoride membranes. Next, membranes were blocked in 5% non‐fat milk, washed for several times and incubated with primary antibodies at 4℃ overnight. NF‐κB, Gasdermin D, caspase‐1, NLRP3, Gapdh and CD81 antibodies were obtained from Cell Signaling Technology (Danvers, MA, USA), and CD63 antibodies were obtained from System Biosciences (Palo Alto, CA, USA). After washed briefly to eliminate unbinding primary antibodies, membranes were incubated with corresponding horseradish peroxidase–conjugated secondary antibodies for 1 hour at room temperature. Finally, proteins were visualized by the enhanced chemiluminescent (ECL) detection systems (Thermo Fisher Scientific).

### 2.14 Data analysis

Statistically significant differences were assessed using the Student's *t* test or one‐way analysis of variance tests in this study. The results were expressed as the mean ± SEM. The criterion for statistical significance was set at *P* <0.05 or *P* <0.01.

## 3 RESULTS

### 3.1 M1 and M2 macrophages showed opposite impact on the LPS‐induced pyroptosis of TCMK‐1 cells in vitro

It is well known that macrophages have a strong plasticity in vivo and in vitro. During the process of AKI, macrophages polarized into different phenotypes in response to the microenvironment and exert diverse effects.^11^, ^12^ In the early stages of AKI, the phenotype of macrophage is mainly M1, which releases the pro‐inflammatory mediator and causes damage to the proximal tubule of the outer layer of the renal medulla. Given the opposite effect of M2 and M1 macrophages, we hypothesized that M2 macrophage may be the key point to prevent the renal damage of AKI.

**FIGURE 1:**
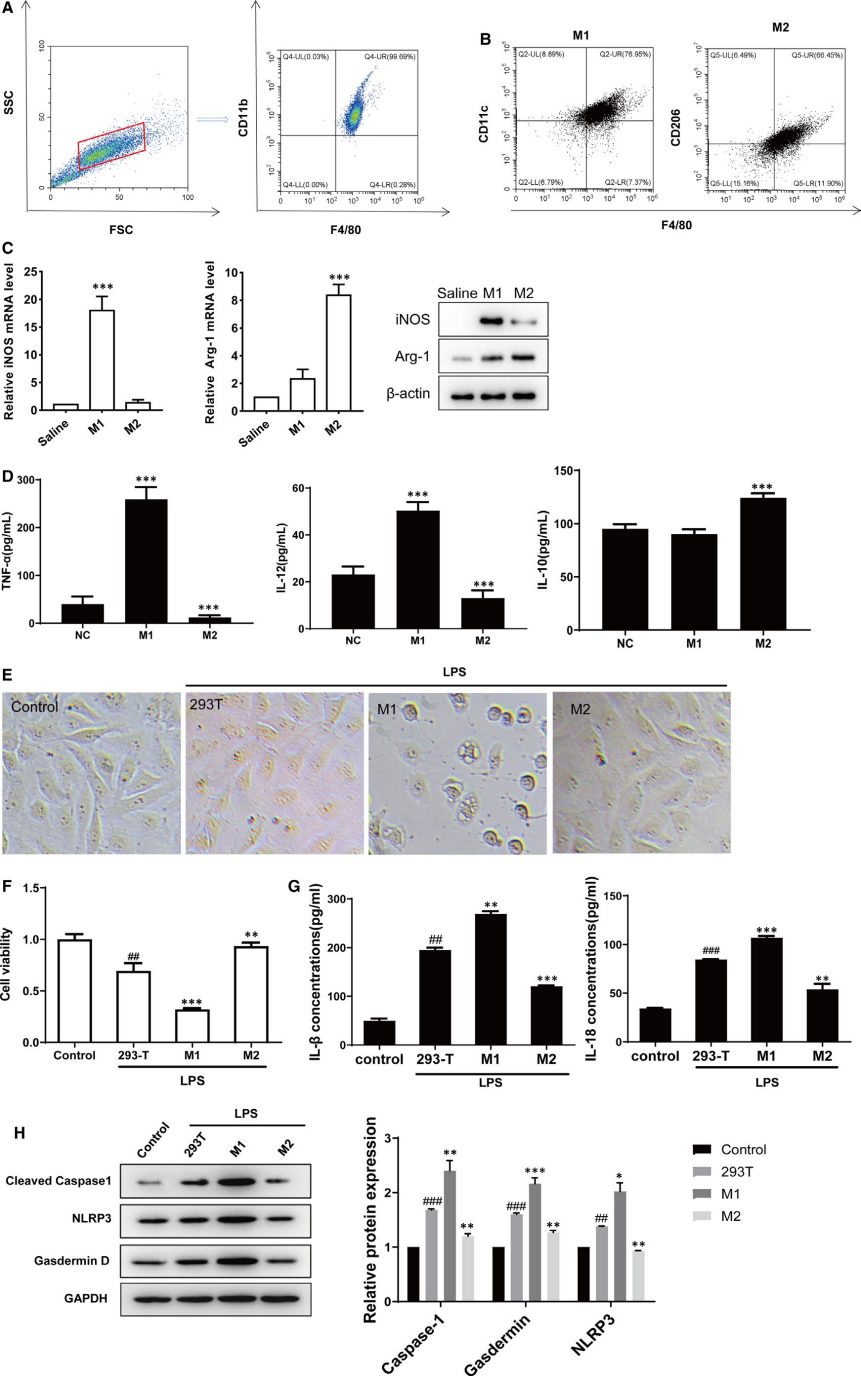
M1 and M2 macrophages showed opposite impact on the LPS‐induced pyroptosis of TCMK‐1 cells in vitro. A, B, The isolated macrophage and polarization of MФ macrophages was identified by flow cytometric analysis with the related biomarkers of M1 macrophages. C, M1 and M2 markers (iNOS and Arg‐1) were detected by using qRT‐PCR and WB. D, ELISA showed that M1 macrophages released TNF‐α and IL‐12, while M2 macrophage released increased IL‐10. E, TCMK‐1 cells were stimulated by LPS for 24 h and then co‐cultured with M1 or M2 macrophages, respectively, for 24 h, using 293T cells as controls. Phase‐contrast imaging assay of TCMK‐1 cell morphology after co‐culture. F, MTT was used to detect the cell viability. G, The concentrations of pyroptosis‐related inflammatory factors IL‐1β and IL‐18 in the culture medium were measured by ELISA. H, Protein expression levels of cleaved caspase‐1, NLRP3 and Gasdermin D were detected by Western blot. Data are presented as mean ± SEM. #*P* <0.05, ##*P* <0.01 and ###*P* <0.001, compared to the control group. *P <0.05, **P <0.01 and ***P <0.001, compared to the 293T group

We then investigated the effect of M1 and M2 macrophages on the LPS‐induced proptosis of TCMK‐1 cells, promoting us to find out the mechanism of the difference effect. We first sorted the macrophages (Figure 1A) and polarization into M1 and M2 macrophages (Figure 1B). M1 macrophage produced more pro‐inflammatory factor TNF‐α and IL‐12 than M2 macrophage, and M2 macrophage released more IL‐10 than M1 macrophage (Figure 1D). We then investigate the effect of M1 and M2 macrophage on LPS‐induced TCMK‐1 injury. M1 and M2 macrophages were, respectively, co‐cultured with TCMK‐1 cells. It was obvious that the TCMK‐1 cells co‐cultured with M1 macrophages showed evident swelling with characteristic large bubbles, indicating the marked cell damage (Figure 1E). MTT assay showed that LPS‐induced significant decrease of cell viability, which was aggregated by co‐culture with M1 macrophage; co‐culture with M2 macrophage inhibited LPS‐induced cell injury (Figure 1F).

Pyroptosis is a new programmed cell death mode which is widely involved in infectious diseases (such as sepsis‐associated AKI). Pyroptosis is mainly characterized by the formation of NLRP3 inflammasomes, which recruit and activate caspase‐1. Caspase‐1 cleaves and activates inflammatory factors such as IL‐18 and IL‐1β, cleaves the N‐terminal sequence of GSDMD and makes it bind to the membrane to generate membrane pores, leading to pyroptosis. We further investigated the effect of M1 and M2 macrophages on cell pyroptosis. The concentrations of IL‐1β and IL‐18 were both higher in the M1 treatment group than in other group (Figure 1G), as well as the expression levels of pyroptosis‐related proteins (Figure 1H). Meanwhile, it could be easily seen that the cell viability, the concentrations of IL‐18 and IL‐1beta and the pyroptosis‐related proteins' level in TCMK‐1 cells co‐cultured with M2 macrophages showed the opposite trend compared with those co‐cultured with M1 macrophages (Figure 1G,H). Hence, we concluded that M1 macrophages could aggravate the level of pyroptosis of TCMK‐1 cells, while M2 macrophages alleviated TCMK‐1 cells on the contrary.

### 3.2 M1‐ and M2‐derived exosomes showed the opposite impact on the pyroptosis in TCMK‐1 cells

**FIGURE 2:**
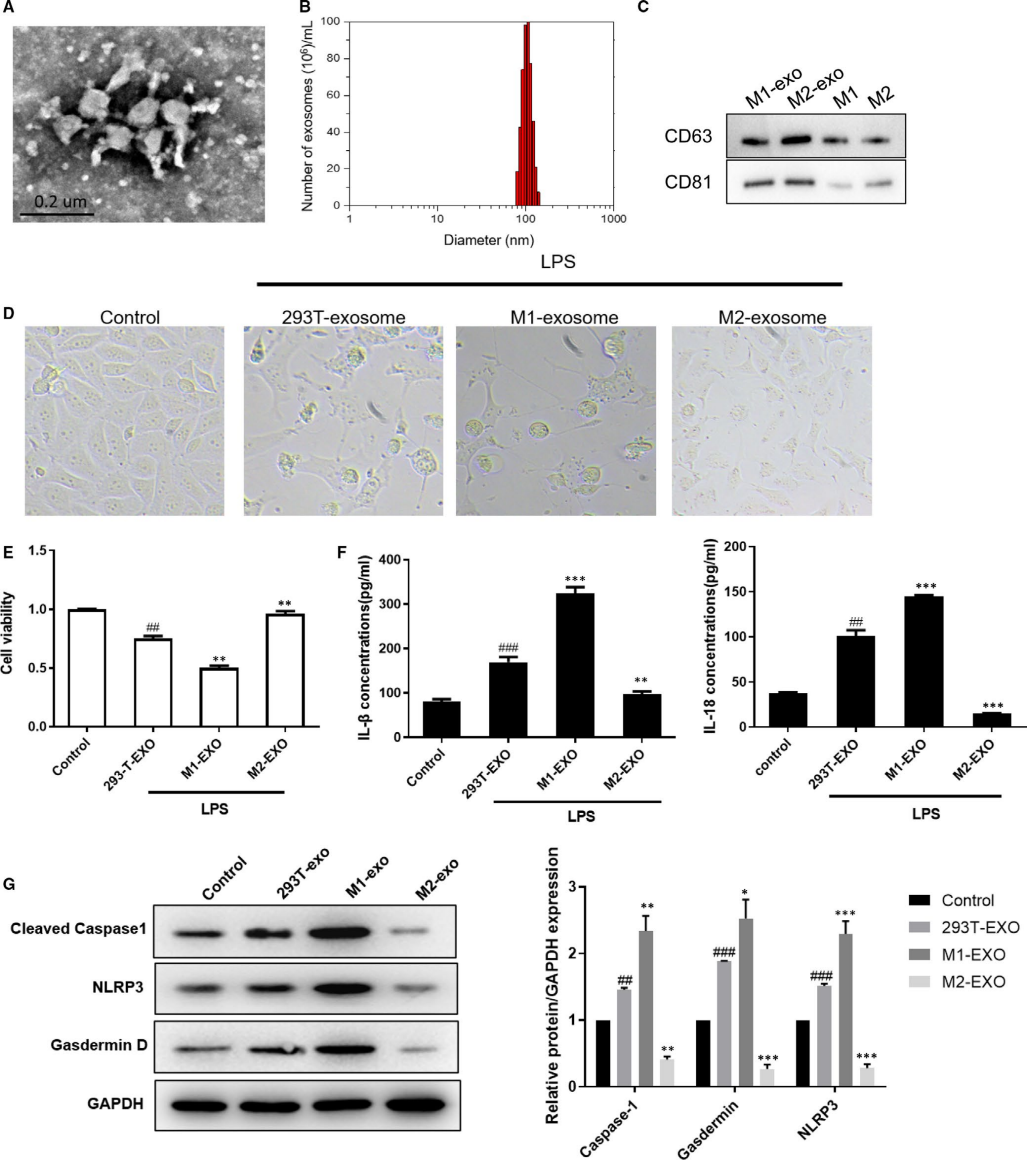
M1‐ and M2‐derived exosomes showed the opposite impact on the pyroptosis in TCMK‐1 cells. The culture media of M1 macrophages, M2 macrophages and 293T cells were collected, where exosomes were extracted. A, Exosomes were analysed under electron microscopy. B, Nanoparticle tracking analysis was used to analyse the size distribution. C, Western blot analysis showing the enrichment of exosome marker of CD63 and CD81. D, Effect of exosomes on LPS‐induced cell morphology was shown. E, MTT was used to detect the effect of exosomes on LPS‐induced cell viability. F, ELISA showing the effect of exosomes on LPS‐induced cell pro‐inflammatory cytokines IL‐1β and IL‐18 release. G, Western blot showing the effect of exosomes on LPS‐induced cell pyroptosis, as indicated by cleaved caspase‐1, NLRP3 and Gasdermin D protein expression. Data are presented as mean ± SEM. ^#^ *P* <0.05, ^##^ *P* <0.01 and ^###^ *P* <0.001, compared to the control group. *P <0.05, **P <0.01 and ***P <0.001, compared to the 293T‐exo group

Exosomes have been found to be the carriers of substances secreted by macrophages to participate in the cellular bioregulation.^28^ We have found the different effect of M1 and M2 macrophages on LPS‐induced TCMK‐1 cells. So, we further investigate the role of exosomes in mediating the effect of M2 macrophage. We extracted exosomes from M1 and M2 macrophages, respectively. Figure 2A shows the typical TEM image of the isolated exosomes. The NTA analysis showed that the particle size distribution in purified pellets measured was consistent with size range of exosomes (average size 100 nm) (Figure 2B). Western blot showed the enrichment of exosome markers CD63 and CD81 of the isolated particles (Figure 2C). TCMK‐1 cells were treated with LPS and then co‐cultured with the extracted exosomes, respectively. As shown in Figure 2D, M1‐derived exosomes enhanced LPS‐induced cell damage of TCMK‐1, while M2‐derived exosomes protected TCMK‐1 from LPS‐induced injury (Figure 2D). MTT assay revealed that M1‐derived exosomes decreased the cell viability, which were recovered by M2‐derived exosomes (Figure 2E). Consistent with the effect of co‐culture with the M1 and M2 macrophages, M1‐derived exosomes induced increased release of IL‐1β and IL‐18, and expression of pyroptosis‐related protein, while M2‐derived exosomes reduced the expression of IL‐1β and IL‐18, and pyroptosis‐related protein, indicating that M1 exosomes promoted cell pyroptosis while M2 exosomes inhibited cell pyroptosis.

### 3.3 miR‐93 exhibited the greatest different expression in macrophage‐derived exosomes, contributing to the inhibition of pyroptosis in TCMK‐1 cells

**FIGURE 3:**
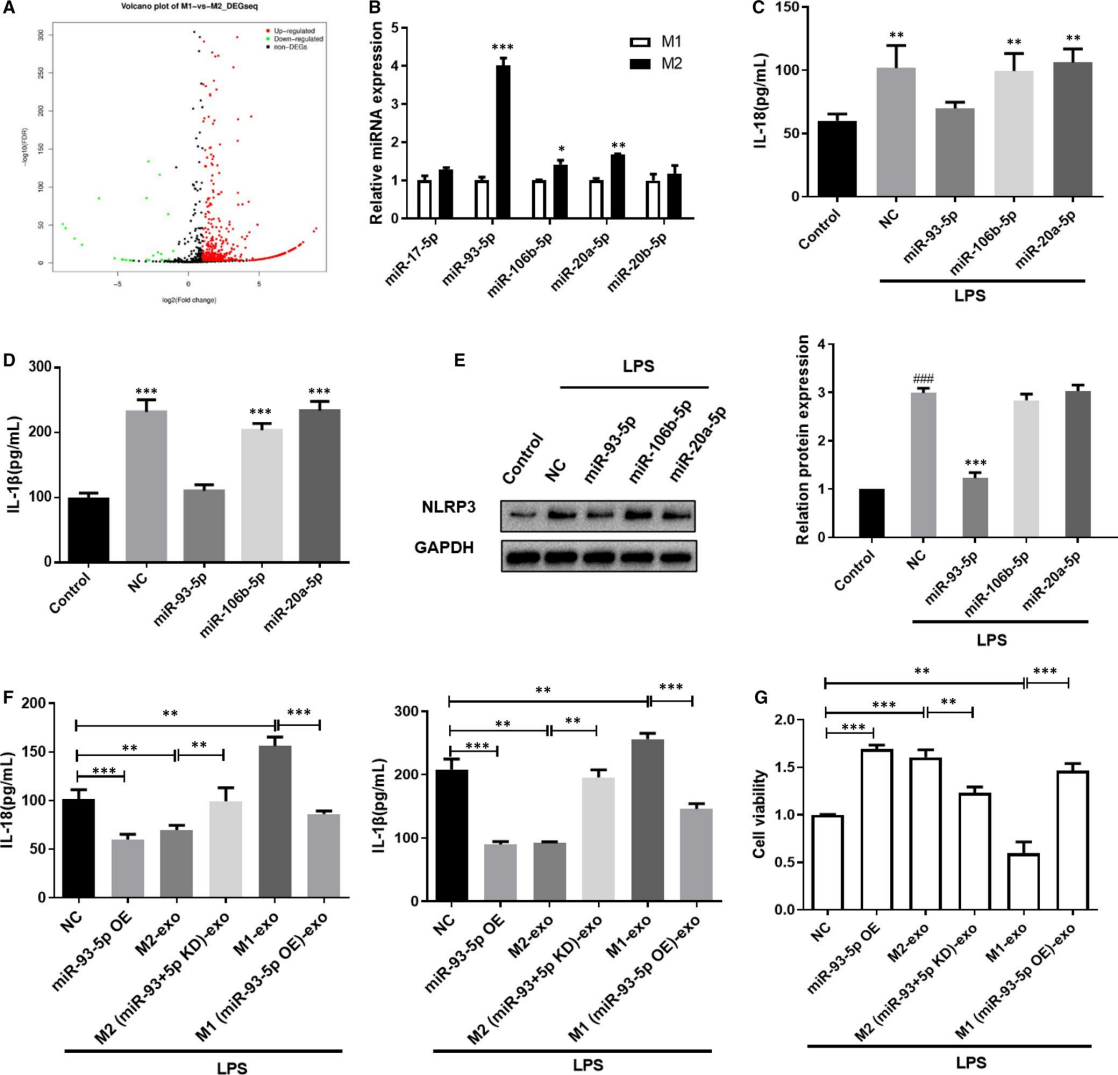
MiR‐93 exhibited the greatest different expression in macrophage‐derived exosomes, contributing to the inhibition of pyroptosis in TCMK‐1 cells. A, Volcano plot showing the distribution of differentially expressed miRNAs in M1 and M2 exosomes. B, qRT‐PCR was used to verify the miRNAs level in M1 and M2 exosomes. C, D, TCMK‐1 cells were treated with miRNA mimic and the indicated control, and then, ELISA was used to measure the IL‐1β and IL‐18 level in the culture medium. E, NLRP3 expression in TCMK‐1 cells was detected by Western blot. F, TCMK‐1 cells were overexpressed with miR‐93‐5p, or treated with M1 and M2 exosomes, and IL‐1β and IL‐18 level in the culture medium was measured by ELISA. G, Cell viability of TCMK‐1 cells in the indicated group was measured by MTT. Data are presented as mean ± SEM. *P < 0.05, **P < 0.01 and ***P < 0.001, compared to the indicated group

Macrophage exosomal miRNAs could play their roles through mediating the biological information among cells.^29^ Here, we examined the differences of miRNAs expression between M1‐ and M2‐derived exosomes (Figure 3A; Table S1). We chose five most differentially expressed miRNAs and verified their expressions in M1 and M2 exosomes by using qPCR. As shown in Figure 3B, miR‐93‐5p, miR‐106b‐5p and miR‐20a‐5p showed the significant expression difference between M1 exosomes and M2 exosomes, with the increased level in M2 exosomes. Besides, we treated the TCMK‐1 cells with miR‐93‐5p mimic, miR‐106b‐5p mimic and miR‐20a‐5p mimic under the LPS induction. We found that miR‐93‐5p markedly reduced the LPS‐induced IL‐18 and IL‐1β expression (Figure 3C,D). Consistently, miR‐93‐5p decreased the LPS‐induced NLRP3 expression (Figure 3E). Therefore, we supposed that exosomal miR‐93‐5p may contribute to the different effect of M1 and M2 macrophages on pyroptosis of TCMK‐1 cells in inflammatory state. We then treated the TCMK‐1 cells with M2 or M2 which knocked down of miR‐93‐5p exosomes, or treated the cells with exosomes from M1 or M1 which overexpressed with miR‐93‐5p. As shown in Figure 3F, we found that overexpression of miR‐93‐5p in cells decreased the LPS‐induced IL‐18 and IL‐1β expression, showing the same effect of M2‐exosomes, while knockdown of miR‐93‐5p abolished the effect of M2‐exosomes; M1 exosomes enhanced LPS‐induced IL‐18 and IL‐1β expression, and exosomes from M1 overexpressed with miR‐93‐5p reduced the IL‐18 and IL‐1β expression. MTT assay showed that treatment of M2 exosomes and overexpression of miR‐93‐5p in cells inhibited the LPS‐induced reduction of cell viability, while knockdown of miR‐93‐5p abolished the effect of M2‐exosomes; M1 exosomes enhanced LPS‐induced decrease of cell viability, and exosomes from M1 overexpressed with miR‐93‐5p recovered the cell viability (Figure 3G).

### 3.4 miR‐93‐5p inhibited pyroptosis pathway in tubular epithelial cells by directly targeting TXNIP

**FIGURE 4:**
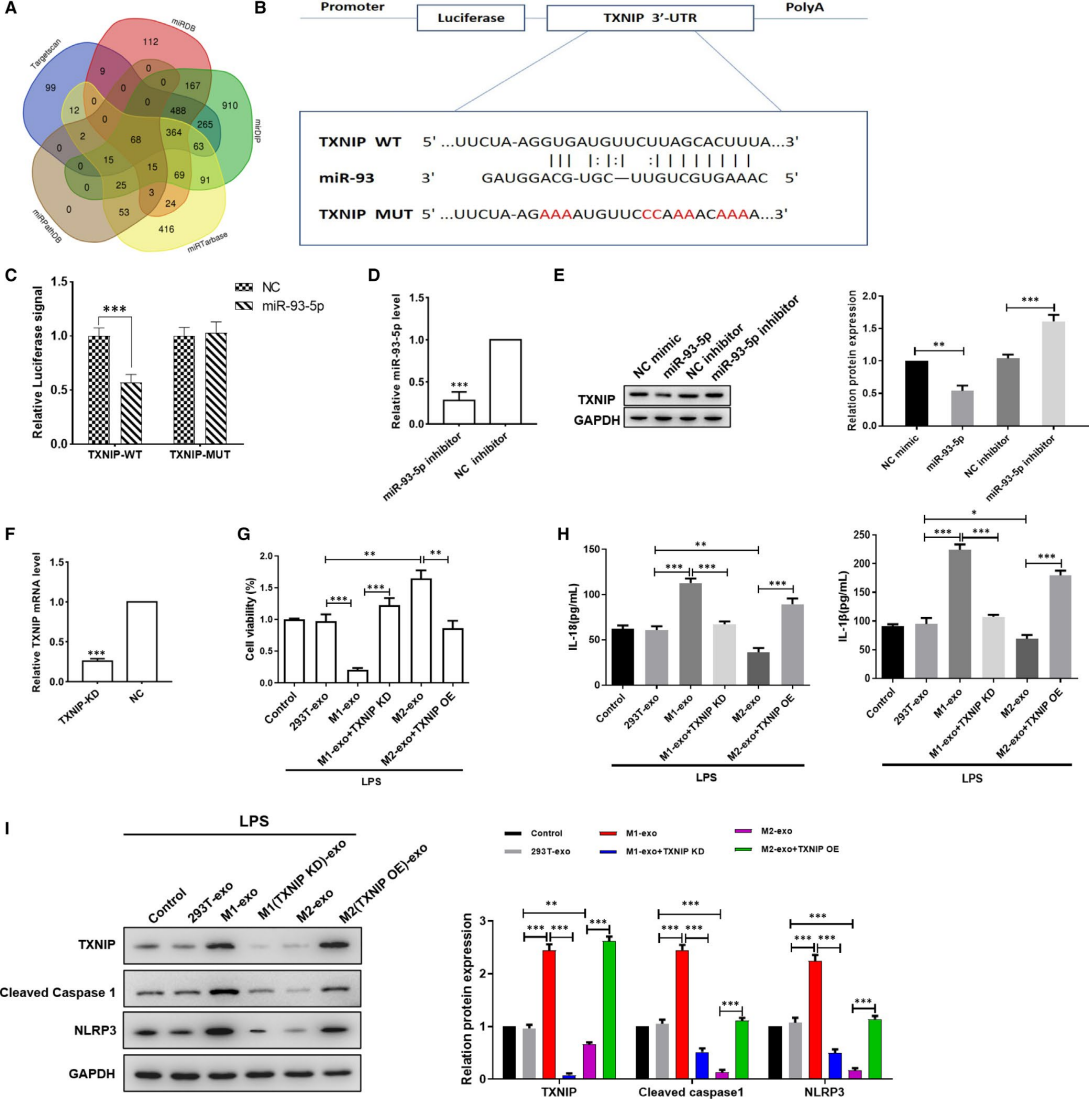
MiR‐93‐5p inhibited pyroptosis pathway in tubular epithelial cells by directly targeting TXNIP. A, Schematic drawing of the screening procedure of the candidate target genes. B, The potential binding sites of miR‐93‐5p in the 3′UTR of TXNIP and the mutant site were shown. C, The luciferase reporter plasmid carrying wild type (WT) or mutant (MUT) TXNIP was transfected into cells with miR‐93‐5p or control mimic. Relative luciferase activity in cells was determined. D, miR‐93‐5p expression level was detected by G. E, Cells were treated with miR‐93‐5p mimic or inhibitor, the expression of TXNIP was determined by Western blot. F, TXNIP expression was level detected by qPCR. G, TCMK‐1 cells were knocked down or overexpressed of TXNIP, with the treatment of M1 and M2 exosomes or not, under the treatment of LPS. Cell viability was measured by MTT. H, Pro‐inflammatory cytokines IL‐1β and IL‐18 level in the culture medium was measured by ELISA. I, Western blot showing the expression of TXNIP, cleaved caspase‐1 and NLRP3 in cells in the indicated group. Data are presented as mean ± SEM. *P < 0.05, **P < 0.01 and ***P < 0.001, compared to the indicated group

We then predicted the downstream target gene by using TargetScan, miRDB, mirDIP, miRTarbase and miRPathDB (Figure 4A). The intersection showed 68 potential target gene, among which we found TXNIP, an activator of NLRP3 pathway. TXNIP is an endogenous inhibitor of the thioredoxin antioxidant and is essential for nod‐like receptor protein 3 (NLRP3) inflammasome activation, which could activate cysteine aspartate protease 1 to regulate the maturation and secretion of IL‐1β and IL‐18 and induce pyroptosis.^30^ The conserved binding sites between miR‐93‐5p and TXNIP are shown in Figure 4B. Luciferase reporter assays demonstrated that co‐transfection of miR‐93‐5p with wild‐type TXNIP luciferase reporter caused a sharp decrease of luciferase activity compared with the mutant TXNIP luciferase reporter, indicating that miR‐93‐5p directly bound to the 3′ UTR of TXNIP (Figure 4C). Western blot further showed that TXNIP protein expression was negatively regulated by miR‐93‐5p mimic and positively regulated by miR‐93‐5p inhibitor (Figure 4E). We then treated the TCMK‐1 cells with M1 or M2 exosomes, with or without knockdown or overexpression of TXNIP. MTT assay showed that M1‐derived exosomes decreased cell viability, which were reversed by knockdown of TXNIP; M2‐derived exosomes increased cell viability, but lost its effect TCMK‐1 cells overexpressed with TXNIP (Figure 4G). M1‐derived exosomes increased the release of IL‐18 and IL‐1β, which were inhibited by knockdown of TXNIP in TCMK‐1 cells (Figure 4H); and M2‐derived exosomes inhibited LPS‐induced IL‐18 and IL‐1β release, while overexpression of TXNIP in TCMK‐1 cells obliterated the effect of M2‐derived exosomes. Moreover, Western blot revealed that the pyroptosis‐related protein showed the same trend in indicated group as the results of IL‐18 and IL‐1β (Figure 4I). These results indicated that the different effect of M1 and M2 exosomes was due to the different activated state of miR‐93‐5p/TXNIP axis.

### 3.5 M2‐derived miR‐93‐5p alleviated AKI through regulating TXNIP

**FIGURE 5:**
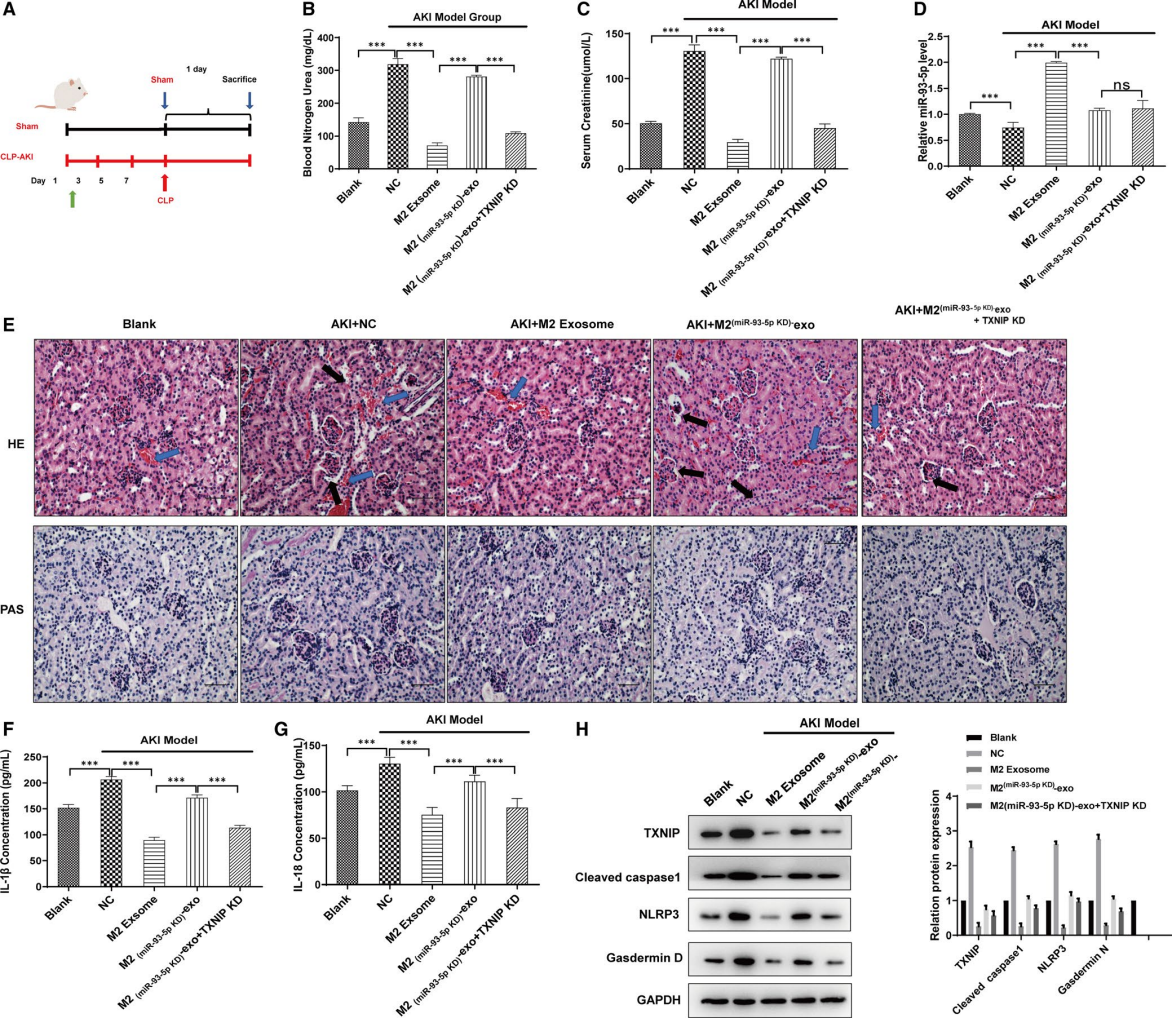
M2‐derived miR‐93‐5p alleviated AKI through regulating TXNIP. Sepsis‐induced AKI model was constructed in mice, and exosomes from M2 or M2 knocked down of miR‐93‐5p were administrated to mice, respectively, with or without TXNIP knockdown by using the knockdown lentivirus. A, We demonstrated the scheme figure to show the process of the animal experiment. B, Blood nitrogen urea and C, serum creatinine of mice in the indicated group was measured by using the commercialized assay kit. D, qRT‐PCR was used to detect the expression of miR‐93‐5p in kidney tissues. E, Kidney tissue sections were subjected to histological examination by H&E and PAS staining to evaluate renal tubule injury. Blue arrows show interstitial congestion and oedema. Black arrows represent intraluminal necrotic cellular debris. F, G, Serum IL‐1β and IL‐18 level was measured by ELISA. G, Western blot showing the expression of TXNIP, cleaved caspase‐1, NLRP3 and Gasdermin D in renal tissues in the indicated group. Data are presented as mean ± SEM. *P < 0.05, **P < 0.01 and ***P < 0.001, compared to the indicated group

**FIGURE 6:**
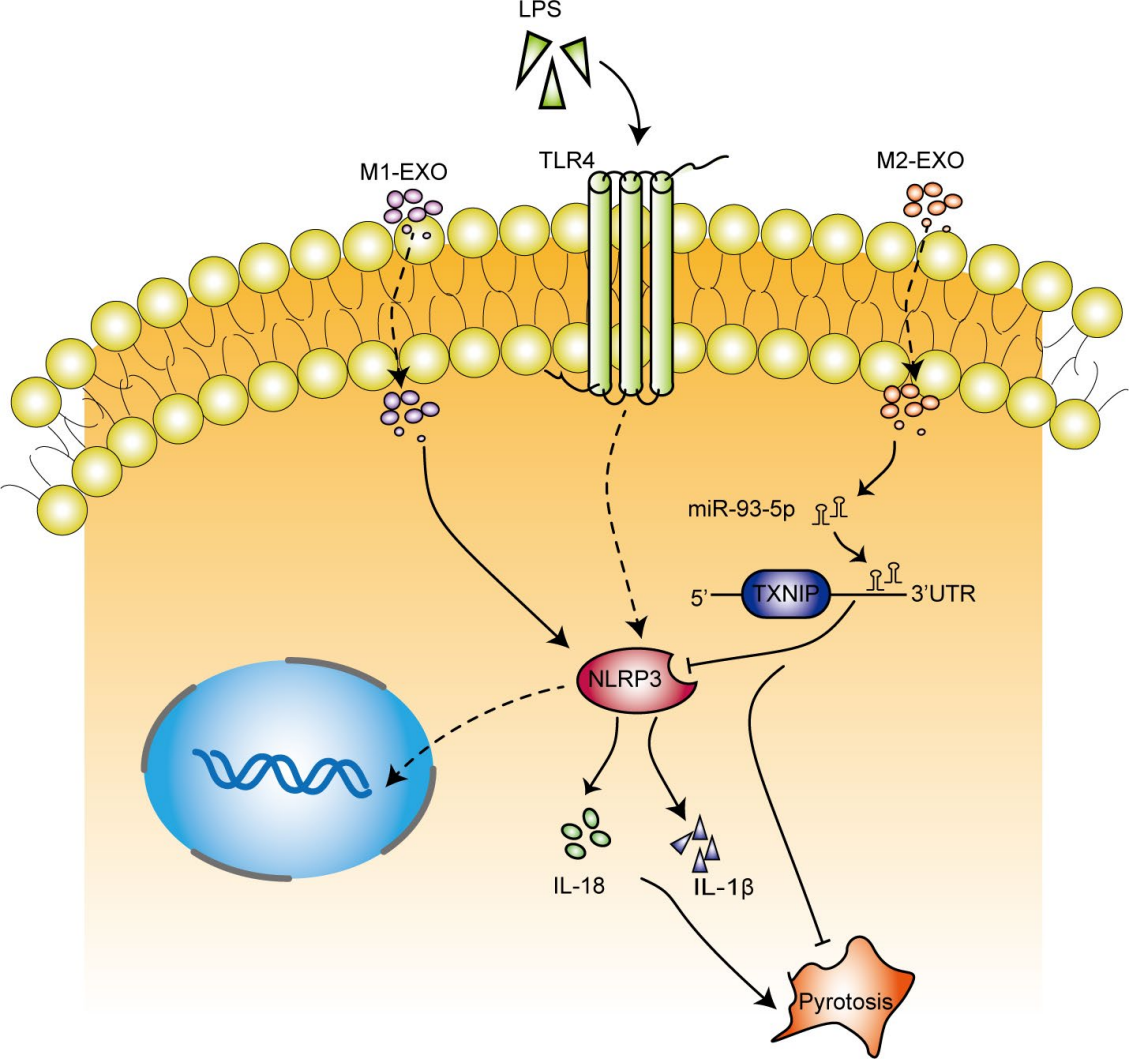
Different phenotypes of macrophages affected the pyroptosis of sepsis‐induced AKI by regulating miR‐93/TXNIP signalling via exosomes delivery

As M2‐derived miR‐93‐5p exhibited obvious protective effect, we wondered if M2‐derived miR‐93‐5p would alleviate AKI in mice. Scheme figure in Figure 5A shows the process of the animal experiment. We constructed the AKI model in mice, and exosomes from M2 or M2 knocked down of miR‐93‐5p were administrated to mice, respectively. We found that M2‐derived exosomes significantly decreased the sepsis‐induced blood nitrogen urea (BUN) concentration and serum creatinine (SCr) concentration, indicating the protective effect of M2 exosomes on the kidney function (Figure 5B,C). Exosomes from M2 knocked down of miR‐93‐5p almost lost their effect on protecting the kidney function (Figure 5B,C). Knockdown of TXNIP in kidney offset the effect of knockdown of miR‐93‐5p in M2, showing a protective effect of kidney function as the M2 exosomes did (Figure 5B,C). qPCR showed that sepsis induced decrease of miR‐93‐5p expression in kidney, while delivery of M2 exosomes significantly increased the miR‐93‐5p level in kidney (Figure 5D). HE staining and PAS staining of the groups showed that the tissue damage was obvious in the AKI model group, but it was milder in the correspond M2‐exosomes‐treated group (Figure 5E). Exosomes from M2 knocked down of miR‐93‐5p could not alleviate the tissue damage, but attenuated the injury with knockdown of TXNIP in kidney (Figure 5F). M2‐derived exosomes significantly decreased the serum IL‐18 and IL‐1β of AKI mice, indicating the inhibition of pyroptosis (Figure 5F,G). Exosomes from M2 knocked down of miR‐93‐5p could not decrease the serum IL‐18 and IL‐1β, and knockdown of TXNIP offset the effect of knockdown of miR‐93‐5p in M2, showing a inhibitory effect of IL‐18 and IL‐1β as the M2 exosomes did (Figure 5F,G). The proteins extracted from kidney tissues showed the same trend as the IL‐18 and IL‐1β, indicating that miR‐93 and TXNIP took an important part in the pyroptosis of kidney tissues in AKI (Figure 5H). Hence, we drew a conclusion that the differences between the function of M1 and M2 macrophages in AKI were caused by miR‐93/TXNIP axis mediated by exosomes (Figure 6).

## 4 DISCUSSION

Previous studies have pointed out that macrophages play important roles in the progress of AKI, and different phenotypes make different contributions at different stages. In the early stage of sepsis, M1 macrophages up‐regulate the expression of pro‐inflammatory mediators, worsening the initial level of tubule injury and augmenting the decrement of glomerular filtration.^31^ While in the late stage, the M1 macrophages transform into M2 macrophages to accelerate the tissue damage repair by promoting the regeneration of the renal tubular epithelium.^32^ In other words, M1 macrophages aggravate sepsis‐induced AKI, while M2 macrophages exhibit protective effect. However, the specific molecular mechanism about the different effect of M1 and M2 macrophages on AKI remains unclear. In the present study, we identified that exosomal miR‐93‐5p was responsible for the different effect of M1 and M2 macrophages, which regulated the cell pyroptosis in sepsis‐induced AKI.

Pyroptosis is a necrotic‐type cell death that was thought to occur exclusively in macrophages,^33^ but recent reports find comparable features in T lymphocytes, neurons and tubular epithelial cells.^34^, ^35^, ^36^ Emerging evidence points out that pyroptosis is involved in the mechanisms for AKI.^37^, ^38^ The inhibition of pyroptosis reduced the inflammatory changes in renal ischaemia/reperfusion injury and decreased the creatinine levels and ameliorated renal dysfunction.^37^ In this study, we demonstrated that M1 and M2 macrophages had opposite influence on the pyroptosis of renal tubular epithelial cells in a co‐cultured system, which might result in the difference between the function of different phenotypes of macrophages in the progress of AKI.

Exosomes are one of the important intercellular communication mechanisms, which could be secreted by damaged cells in many pathologic conditions to alter the phenotype of targeted cells by transferring microRNA, mRNA and protein‐based transcription factors.^39^ In the present study, we found that the pyroptosis level of TCMK‐1 cells co‐cultured with only exosomes extracted from macrophages showed similar trend with co‐cultured with macrophages themselves, indicating that M1 and M2 macrophages may regulate the viability of renal tubular epithelial cells through delivering some functional molecules. Studies have suggested that microRNAs are involved in a variety of biological processes, which includes sepsis‐induced AKI. For example, miR‐124, miR‐204 and miR‐107 have been found mediate the sepsis‐induced AKI by specifically inhibiting the translation of target genes.^40^, ^41^, ^42^ So, we examined the differentially expressed microRNAs in exosomes extracted from M1 and M2 macrophages, respectively, via high‐throughput sequencing to investigate whether miRNAs are involved in the different effect of M1 and M2 macrophages. The results revealed that the expression of miR‐93‐5p showed the greatest difference, with a significant increase in the M2‐derived exosomes. And further experiments found that knockdown of miR‐93‐5p in M2 macrophages obliterated the effect of M2‐derived exosomes, while overexpression of miR‐93‐5p in M1 macrophages obliterated the pro‐inflammatory effect of M1‐derived exosomes, demonstrating that exosomal miR‐93‐5p led to the different effect between M1 and M2 macrophages on the pyroptosis level of renal tubular epithelial cells.

TXNIP was reported to interact with accumulated reactive oxygen species and stimulate inflammation and cell apoptosis.^31^, ^43^, ^44^ Increasing studies have pointed out that the combination of TXNIP and inflammasome plays a causative role in ischaemia/reperfusion,^45^, ^46^ which is a critical risk factor for AKI. The knockdown of TXNIP could significantly inhibit the activation of NLRP3 inflammasome in I/R injured HK‐2 cells as characterized by decreased IL‐1β and IL‐18 levels, which represent the level of pyroptosis.^43^ TXNIP is suggested as an important future target to develop newer therapeutics for its ability to reduce AKI sensitivity of kidney tissues.^45^ In the present study, we identified the potential targets of miR‐93‐5p and found that TXNIP was a candidate target of miR‐93‐5p. Following luciferase reporter assay and western blot further verified that miR‐93‐5p negatively regulated the TXNIP expression through binding to its 3′UTR. Overexpression of TXNIP significantly abolished the effect of miR‐93‐5p on restraining the cell pyroptosis. Our findings indicated that exosomal miR‐93 excreted from macrophages exerted its regulatory role on TXNIP in TCMK‐1 cells. To confirm that our hypothesis also makes sense in vivo, we generated the mouse model of sepsis‐induced AKI by CLP. The results proved that exosomal miR‐93‐5p inhibited the pyroptosis during AKI progression through regulating TXNIP.

In summary, the present study demonstrated that different phenotypes of macrophages affected the pyroptosis of sepsis‐induced AKI by regulating miR‐93/TXNIP signalling via exosomes delivery. Therefore, exosomal miR‐93/TXNIP signalling plays a crucial role in the progress of sepsis‐induced AKI, which provides potential targets for the treatment of AKI.

## CONFLICT OF INTEREST

The authors confirm that there is no conflict of interests.

## AUTHOR CONTRIBUTIONS

Chen‐Xia Juan: Conceptualization (lead); Formal analysis (lead); Investigation (lead); Methodology (lead); Software (equal); Supervision (equal); Validation (lead); Visualization (lead); Writing‐original draft (lead). Yan Mao: Conceptualization (supporting); Data curation (lead); Formal analysis (supporting); Writing‐review & editing (lead). Qian Cao: Data curation (supporting); Investigation (supporting); Methodology (lead); Project administration (supporting); Software (supporting). Yan Chen: Methodology (supporting); Resources (lead); Supervision (supporting). Lan‐Bo Zhou: Validation (supporting); Visualization (supporting); Writing‐review & editing (supporting). Sheng Li: Data curation (supporting); Formal analysis (supporting); Resources (supporting); Software (supporting); Writing‐review & editing (supporting). Jiahe he Chen: Investigation (supporting); Project administration (supporting). Hao Chen: Conceptualization (supporting); Data curation (supporting); Formal analysis (supporting); Methodology (supporting); Resources (supporting). Guo‐Ping Zhou: Conceptualization (supporting); Data curation (supporting); Formal analysis (supporting); Funding acquisition (lead); Investigation (supporting); Methodology (supporting). Rui Jin: Project administration (lead); Resources (supporting); Software (supporting); Supervision (supporting); Validation (supporting); Visualization (supporting); Writing‐original draft (supporting).

## Supporting information

Table S1

## Data Availability

The data that support the findings of this study are available from the corresponding author upon reasonable request.
