# Supplementary material for: Exosome‐mediated pyroptosis of miR‐93‐TXNIP‐NLRP3 leads to functional difference between M1 and M2 macrophages in sepsis‐induced acute kidney injury
Source: J Cell Mol Med. 2021 Mar 21;25(10):4786–99. doi: 10.1111/jcmm.16449 (PMC8107088; doi:10.1111/jcmm.16449)
Supplement: Supplementary file 1 — Table S1 [file JCMM-25-4786-s001.docx]

| **miRNA id** | **Expression(M1)** | **Expression(M2)** | **log2Ratio(M2/M1)** |
| --- | --- | --- | --- |
| mmu-miR-93-5p | 806.7266667 | 2978.55 | 2.08064 |
| mmu-miR-106b-5p | 6912.173333 | 20734.76333 | 1.76979 |
| mmu-miR-20a-5p | 135.5966667 | 349.4533333 | 1.558632 |
| mmu-miR-20b-5p | 0.106667 | 2.85 | 4.888237 |
| mmu-miR-17-5p | 387.0633333 | 912.5033333 | 1.433100475 |
| mmu-miR-652-3p | 3885.877 | 7907.957 | 1.221249 |
| mmu-miR-7a-5p | 9183.413 | 17832.67 | 1.141707 |
| mmu-let-7d-5p | 7615.67 | 14980.44 | 1.16938 |
| mmu-miR-96-5p | 752.0133 | 1414.903 | 1.10481 |
| mmu-miR-320-3p | 387.0633 | 912.5033 | 1.4331 |
| mmu-miR-340-5p | 468.2567 | 985.4867 | 1.267352 |
| mmu-miR-181d-5p | 562.61 | 1098.947 | 1.160925 |
| mmu-miR-26b-5p | 780.43 | 1509.503 | 1.146003 |
| mmu-miR-96-5p | 752.0133 | 1414.903 | 1.10481 |
| mmu-miR-17-3p | 113.8633 | 260.9067 | 1.389821 |
| mmu-miR-872-5p | 184.65 | 350.86 | 1.124258 |
| mmu-miR-340-3p | 28.62 | 66.51667 | 1.41132 |
| mmu-miR-193a-5p | 45.32667 | 88.33667 | 1.160244 |
| mmu-miR-142b | 47.15333 | 90.61667 | 1.13288 |
| mmu-miR-505-5p | 28.93333 | 59.36667 | 1.227271 |

**Supplementary Table 1 20 upregulated miRNAs in the M2 exosomes**
